# NanoBioAccumulate: Modelling the uptake and bioaccumulation of nanomaterials in soil and aquatic invertebrates via the Enalos DIAGONAL Cloud Platform

**DOI:** 10.1016/j.csbj.2024.09.028

**Published:** 2024-10-17

**Authors:** Dimitris G. Mintis, Nikolaos Cheimarios, Andreas Tsoumanis, Anastasios G. Papadiamantis, Nico W. van den Brink, Henk J. van Lingen, Georgia Melagraki, Iseult Lynch, Antreas Afantitis

**Affiliations:** a NovaMechanics Ltd., Nicosia 1070, Cyprus; b Entelos Institute, Larnaca 6059, Cyprus; c School of Geography, Earth and Environmental Sciences, University of Birmingham, Birmingham B15 2TT, United Kingdom; d Department of Toxicology, Wageningen University, Wageningen, the Netherland; e Laboratory of Systems and Synthetic Biology, Wageningen University & Research, Wageningen, the Netherland; f Division of Physical Sciences and Applications, Hellenic Military Academy, Vari 16672, Greece; g NovaMechanics MIKE, Piraeus 18545, Greece

**Keywords:** Nanomaterials, Nanomaterial bioaccumulation, Biokinetic models, Invertebrates, Web application, Enalos diagonal cloud platform, Nonlinear regression, Genetic algorithm

## Abstract

*NanoBioAccumulate* is a free-to-use web-based tool hosted on the Enalos DIAGONAL Cloud Platform (https://www.enaloscloud.novamechanics.com/diagonal/pbpk/) that provides users with the capability to model and predict the uptake and bioaccumulation of nanomaterials (NMs) by soil and aquatic invertebrates using two common first-order one-compartment biokinetic models. *NanoBioAccumulate* offers an approach for comprehensively analyzing the kinetics of different forms of NMs via a nonlinear fitting feature, integrating them with environmental fate models, and considering important physiological processes. *NanoBioAccumulate* overcomes the constraint of requiring prior knowledge of kinetic rate constants associated with the biokinetic models and eliminates the need for external statistical analysis software as it quantifies the kinetic rate constants and other constants through the application of nonlinear regression, using user-provided experimental data. Furthermore, *NanoBioAccumulate* incorporates statistical analysis measures like the adjusted R-squared and the bias-corrected Akaike information criterion, allowing for assessment of the goodness-of-fit of the two different biokinetic models, assisting in the identification of the best-performing model for a specific nanoform and its uptake kinetics by a specific invertebrate. The tool also includes model scenarios, retrieved from literature, which involve examining the exposure of soil and aquatic invertebrates to various types of NMs such as TiO_2_, SiO_2_, C_60_, graphene, graphene oxide (GO), Au, Ag and its ionic control AgNO_3_. These model scenarios aim to enhance understanding of the uptake and elimination rates exhibited by different NM-species. *NanoBioAccumulate* features advanced integration capabilities, enabled by an extensive Application Programming Interface (API). This functionality promotes efficient data exchange and interoperability with other software and web applications, significantly expanding its utility in research, regulatory risk assessment and environmental surveillance and monitoring contexts. The inclusion of a user-friendly Graphical User Interface (GUI) in *NanoBioAccumulate* greatly improves the overall user experience by simplifying complex tasks and eliminating the need for programming proficiency, thereby expanding the tool's applicability to a diverse range of users across various fields such as environmental research, monitoring, and regulation.

## Graphical Abstract

**Figure:**
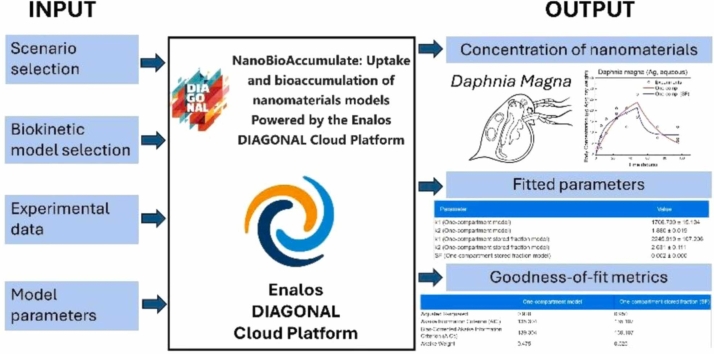

## 1 Introduction

In recent years, the rapid growth of nanotechnology has resulted in significant advances across various sectors, such as biotechnology, food production, energy, textiles, optoelectronics and catalysis. [1], [2], [3], [4], [5], [6] Nanotechnology has become increasingly prominent within the computer industry, particularly in response to the growing demand for the production of smaller and faster transistors, to effectively reduce the size and cost of computers while significantly enhancing their operational speed. [6] Since their size is similar to that of biological macromolecules (in the size range of 1 nm-100 nm) [7], [8] and because of their antibacterial and odor-fighting properties, [6] engineered nanomaterials (NMs) are extensively used in a number of commercial products such as sunscreens, cosmetics, plastics, paints, wound dressing, detergents and antimicrobial coatings. [6], [7] Silver (Ag), zinc oxide (ZnO), titanium dioxide (TiO_2_), silicon dioxide (SiO_2_), graphene oxide (GO), fullerene (C_60_), and cerium dioxide (CeO_2_) are among the most commonly manufactured metal and metal oxide-based NMs. [9].

The widespread application and the rapid production of large amounts of engineered NMs presents notable environmental concerns, especially when these NMs enter terrestrial and aquatic ecosystems. [10], [11], [12] NMs have already been identified as emerging contaminants of environmental concern (CEC) in numerous international reports. [13] Once released, NMs pose a complex risk to environmental health, primarily through their interactions with, and uptake by, living organisms. This interaction is not merely a localized hazard; it extends to a broader ecological scale, potentially impacting human health via the food chain. [14] The bioaccumulation of these particles in terrestrial and aquatic life can lead to their incorporation into the human diet, thereby introducing unknown risks to human health. [12] Understanding these pathways is crucial for assessing the full scope of nanotechnology's environmental footprint and safeguarding against potential long-term consequences. However, investigating the interactions between NMs and aquatic and terrestrial ecosystems poses challenges due to the experimental complexities associated with studying particle uptake at environmentally applicable concentrations, typically in the range of μg L^−1^ or μg kg^−1^. [14] Notably, the data used for the calibration and validation of the web application developed in this work (as discussed in Section 6) were obtained from previously published, peer reviewed experimental studies, which employed well-established experimental techniques including inductively coupled plasma mass spectrometry (ICP-MS) and, transmission electron microscopy (TEM) to accurately quantify NMs uptake and bioaccumulation in invertebrates, and dynamic light scattering (DLS), and UV–visible spectrophotometry to characterize the nanomaterials before and during the exposures. [15], [16], [17], [18], [19].

Though there has been a strong focus so far on the toxic effects of NMs, [20] exposure assessment research for modelling the uptake and accumulation of NMs in terrestrial and aquatic organisms is lagging. [13], [21] Various modelling approaches have been suggested for predicting the uptake of NMs by organisms, including the use of i) Accumulation Factors employed commonly for organic compounds (such as the Bioaccumulation Factor (BAF), the Bioconcentration Factor (BCF) and the Biomagnification Factor (BMF)), [22], [23], [24], ii) biotic ligand models (BLMs) for modelling metal uptake, [25], [26] and iii) biokinetic models that utilize uptake and elimination kinetic parameters to simulate contaminant accumulation. [13], [27] Although the implementation of Accumulation Factors is commonly used and has been widely adopted in various studies due to its relative simplicity, [28], [29], [30], [31], [32] it is important to note that the assumption of steady state is not always valid in NMs studies, hence this approach should be cautiously employed. [28], [29], [33] BLMs, are constrained in their ability to accurately simulate the uptake and toxicity of NMs compared to ionic metals, as they are designed for short-term exposures usually lasting between 24 to 96 h and can only assess acute effects. [13], [34] The Michaelis-Menten approach which is employed in some studies [35], [36] to quantify the uptake rates of NMs to organisms in short-term exposures is considered to be a similar approach to BLMs. [37] In contrast, biokinetic models have the potential to characterize and predict the biokinetics of NMs over a longer time frame, thereby facilitating the establishment of more robust and accurate long-term bioaccumulation models. [30], [32], [35], [36], [38], [39], [40], [41].

Dynamic biokinetic models offer a systematic approach to understanding the biokinetics of NMs in terrestrial and aquatic organisms by taking into account the physicochemical properties and other relative factors of NMs. [37] These models facilitate deeper understanding and predictive power regarding the uptake and accumulation patterns of NMs in organisms. [30], [37] Through the application of toxicokinetic parameters, biokinetic models quantify the rates at which NMs are taken up (absorbed) and eliminated by organisms, allowing for precise predictions of their biokinetics. [32], [35], [36] These models consider the transfer of NMs from one compartment (such as water or soil) to organisms, and vice versa, [13] and their applicability in environmental risk assessments can be further broadened by including environmental elimination mechanisms such as sedimentation. [42], [43].

**Fig. 1:**
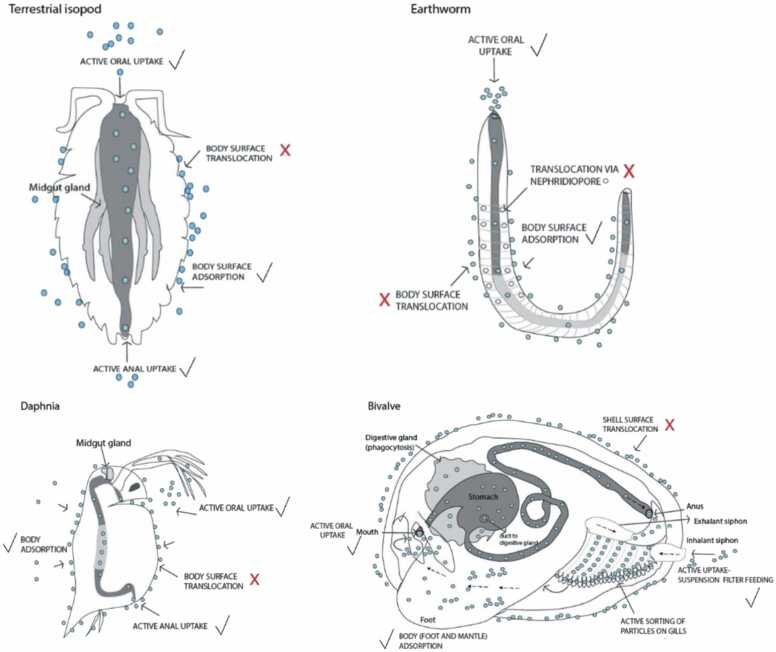
Active and passive uptake of NMs in different representative invertebrate species, commonly used in environmental research: terrestrial isopod (Crustacea), earthworm (Annelida), water flea (Crustacea), and bivalve (Mollusca; e.g., Mytilus sp.; only one valve with a removed mantle is shown). The organisms are presented schematically with a focus on the guts (as the major uptake route), but the position and length of each gut region is not shown at realistic scales. NMs are shown as blue circles. Routes where NMs passage is questionable or unproven are marked with a red cross. Probable uptake routes are marked with a black tick symbol. Reproduced from the work of Van den Brink et al. [37] Note that the models implemented in the NanoBioAccumulate web application do not take into account the adsorption mechanism for uptake.

The majority of research studies consider soil or aquatic invertebrates as the organism of choice for evaluating the environmental risks posed by NMs. [18], [21], [35], [36], [38], [40], [41], [44], [45], [46], [47] This is attributed to the high diversity of invertebrates, representing over 99 % of all animal species, and their crucial role in various ecological functions such as soil structure maintenance and nutrient cycling, which are closely interconnected with ecosystem services. [37], [48], [49] Fig. 1, reproduced from the work of Van den Brink et al., [37] illustrates the possible routes of active and passive uptake of NMs (shown as blue circles) in different invertebrate species, namely of terrestrial isopod (Crustacea), earthworm (Annelida), water flea (Crustacea), and bivalve (Mollusca). The gut of each species is shown as a grey tube. As can be observed in Fig. 1, the primary route of NMs uptake in invertebrates is actively via ingestion and to a comparatively smaller extent by anal uptake, as well as passive uptake through body surfaces or body openings. [37], [50], [51] Other potential routes of active uptake of NMs include uptake of water [52] and potential adsorption onto the body surfaces of organisms, as illustrated in Fig. 1. [53], [54], [55].

Developing user-friendly web interfaces for studying nanotoxicology and nanosafety is essential in making complex scientific data accessible and comprehensible to a broader audience, including researchers and stakeholders with no coding experience. Simplifying the interaction with complex datasets and analytical tools through well-designed Graphical User Interfaces (GUI) contributes to the widespread dissemination of knowledge and the enhancement of collaborative research efforts. A well-designed GUI allows for intuitive navigation and interaction with the software, making complex tasks more manageable and efficient, thus broadening the potential user base for the modelling tool. Furthermore, the integration through Application Programming Interfaces (APIs) is vital for the integration of different software systems, facilitating the efficient exchange of data and enhancing the functionality and interoperability of scientific tools. APIs enable diverse applications to communicate and work together, thereby expanding the capabilities and applications of individual tools. In the context of nanosafety for example, this has been demonstrated through integration of NMs exposure and biodistribution models into an Integrated approach to Testing and Assessment (IATA), which was further integrated with a hazard prediction model. [56].

The application of dynamic biokinetic models in understanding the biokinetics of NMs bioaccumulation in terrestrial and aquatic organisms holds significant potential for advancing efficiency in risk assessment and the implementation of Safe and Sustainable by Design (SSbD) strategies. However, a primary challenge persists in accurately quantifying kinetic rate constants for determining uptake rates for different NM species, as well as their excretion rates and rates of dissolution in the environment. In response to this challenge, we introduce *NanoBioAccumulate*, a user-friendly web application designed specifically for modelling the biokinetics of the whole-body accumulation of NMs in soil and aquatic invertebrate organisms. Modelling the tissue-specific uptake patterns and internal distribution in soil and aquatic invertebrate organisms is out of the scope in this study due to the limited literature. The web application includes two biokinetic models: (i) a simple one compartment (OC) model that considers the organism as a single compartment, with just uptake and elimination as kinetic processes; and (ii) a one compartment model considering the addition of a stored fraction (SF) of NMs or their dissolved fraction, called the one compartment with stored fraction (OC-SF) model. The *NanoBioAccumulate* web application utilizes nonlinear regression to fit user-provided experimental data to quantify kinetic rate constants and other constants (such as the SF) of the biokinetic models. Additionally, *NanoBioAccumulate* features scenario modelling to simulate the uptake and accumulation of NMs in diverse soil and aquatic invertebrates, leveraging prior reported data to build a dataset that gives insights into the potential risks of various NMs to these organisms.

## 2 Biokinetic models integrated in NanoBioAccumulate

The dynamic biokinetic models incorporated into the *NanoBioAccumulate* web application are presented in this section. *NanoBioAccumulate* implements the one-compartment (OC) model and the one-compartment model with a stored fraction (OC-SF) that accounts for the NMs stored or accumulated in the bodies of the organisms during the uptake and elimination phases.

### 2.1 One compartment (OC) model

In the simplest model, the organism is treated as a single compartment, allowing for the estimation of one uptake rate constant and one elimination rate constant. This model uses (1), (2) to describe the uptake and elimination of the NMs, respectively, during the exposure period.

(1) $$C_{org^{t}}=C_{org^{t=0}}+C_{\mathrm{exposure}}\times \left(\frac{k_{1}}{k_{2}}\right)\times \left(1-e^{\left(-k_{2}\times t\right)}\right),0\leq t\leq t_{e}$$

(2) $$C_{org^{t}}=C_{org^{t=0}}+C_{\mathrm{exposure}}\times \left(\frac{k_{1}}{k_{2}}\right)\times \left(e^{\left(-k_{2}\times \left(t-t_{e}\right)\right)}-e^{\left(-k_{2}\times t\right)}\right),t>t_{e}$$

where $C_{org^{t}}$ is the concentration in the organism at time t (mg kg^−1^), $C_{org^{t=0}}$ is the concentration in the organism at t=0 (mg kg^−1^ in soil), $C_{\mathrm{exposure}}$ is the concentration in the exposure medium (mg kg^−1^ or mg L^−1^), $k_{1}$ is the uptake rate constant (kg_medium_ kg_organism_^−1^ per day or per hour or L_medium_ kg_organism_^−1^ per day or per hour), $k_{2}$ is the elimination rate constant (day^−1^ or hour^−1^), t is the time (day or hour), $t_{e}$ is the time at which the test organisms are transferred from a contaminated to a clean medium (day or hour).

### 2.2 One compartment model with a stored fraction (OC-SF)

The accumulation and storage of nanoparticles (NPs) within the bodies of organisms is observed to vary depending on several factors, including the physiological characteristics of the organisms, the medium concentration, the characteristics of the nanoparticles, and the route of exposure. [37], [45] For instance, studies have shown that isopods store Ag from Ag-NMs and AgNO_3_, as well as Co from CoFe_2_O_4_ NMs, in their digestive glands or hepatopancreas. [40], [57] In the case of earthworms, it was previously reported that metals could potentially be stored in the chloragogenous tissue (i.e. star-shaped cells involved with excretory functions and intermediary metabolism in annelids), which contains phosphorus, calcium, and sulfur. [58] As was shown by Ribeiro et al., [18] who assessed the uptake of Ag-NMs by *D. magna*, this storage may be applicable to NMs or to the metal ions released from metal oxide based NMs. Some studies, [18], [40], [59] considered storage only in the elimination phase and did not take it into account in the uptake phase when the organisms were exposed to NMs. However, in our study, we consider storage in both the uptake and elimination phases.

To account for this stored accumulation factor during both the uptake and elimination phases, (1), (2) can be extended with the consideration of a dimensionless stored fraction, SF, as described below in (3), (4):

(3) $$C_{org^{t}}=C_{org^{t=0}}+\mathrm{SF}\times C_{\mathrm{exposure}}\times k_{1}\times t+\left(1-\mathrm{SF}\right)\times C_{\mathrm{exposure}}\times \left(\frac{k_{1}}{k_{2}}\right)\times \left(1-e^{\left(-k_{2}\times t\right)}\right),0\leq t\leq t_{e}$$

(4) $$C_{org^{t}}=C_{org^{t=0}}+\mathrm{SF}\times C_{\mathrm{exposure}}\times k_{1}\times t_{e}+\left(1-\mathrm{SF}\right)\times C_{\mathrm{exposure}}\times \left(\frac{k_{1}}{k_{2}}\right)\times \left(e^{\left(-k_{2}\times \left(t-t_{e}\right)\right)}-e^{\left(-k_{2}\times t\right)}\right),t>t_{e}$$

where SF is the stored fraction, taking a value between 0 and 1.

## 3 Statistical analysis integrated into NanoBioAccumulate

### 3.1 Nonlinear regression analysis

To be able to predict the patterns of uptake and elimination of NMs by organisms, knowledge of the kinetic parameters *k*_1_ and *k*_2_ and of the SF parameter is crucial. Nonlinear regression is commonly used to fit experimental data regarding the concentration of NMs in the body of organisms over time as a means to quantify these parameters. [18], [19], [37], [46] However, users who do not have coding experience often rely on the use of commercial statistical analysis software such as GraphPad Prism, [60] JMP, [61] SPSS, [62] Minitab, [63] Statgraphics Centurion, [64] Genstat, [65] Statistica, [66] Isalos, [67] and other similar tools.

The *NanoBioAccumulate* web-tool offers an alternative solution by providing users with the ability to perform nonlinear regression fitting on user-provided experimental data, eliminating the need for commercial software. This allows users to quantify the kinetic parameters and the SF parameter and make predictions about NMs uptake and elimination patterns in organisms. Though, alternative free-to-use software packages such as in R exist for this purpose, the advantage of the *NanoBioAccumulate* is that it is a free-to-use web-based tool that doesn’t require local compilation or installation and can be accessed from any location that has access to a web browser.

*NanoBioAccumulate* utilizes the genetic algorithm approach for nonlinear regression and integrates the Jenetics library [68] in Java. This library includes components such as Genotype, DoubleGene, and DoubleChromosome, which define the genetic structure of solutions. The Engine, EvolutionResult, and Limits components are used for building and running the genetic algorithm. Other components like Mutator and SinglePointCrossover perform genetic alterations, while RandomRegistry ensures reproducibility by controlling randomness.

To configure the genetic algorithm engine, a population size of 1000 is set, and Mutator and SinglePointCrossover alterers are employed to maintain genetic diversity. Single-threaded execution guarantees reproducibility, and a steady fitness limit of 300 generations serves as the stopping condition for the algorithm.

The selection of the genetic algorithm (using the Jenetics library) was rather complex and was a result of a thorough evaluation of various nonlinear regression techniques, including the Gradient Descent algorithm and the Levenberg-Marquardt algorithm (utilizing the Apache commons math library). [69], [70], [71] Despite the simplicity, in terms of programming and design, of the Gradient Descent and the Levenberg-Marquardt algorithms, when compared to the genetic algorithm, these alternative approaches were not capable of achieving high correlations in terms of goodness-of-fit with the experimental data points. Furthermore, the calculated fitted values obtained using these methods did not align closely with the previously reported calculations (in the paper of Zheng and Nowack), [46] which utilized the GraphPad Prism software. In contrast, the genetic algorithm demonstrated superior performance, as evidenced by the close agreement between the fitted values generated by our tool and those reported by Zheng and Nowack, [46] as detailed in Table 1.

**Table 1:** Comparison between results generated using the web-tool NanoBioAccumulate and results obtained from the work of Zheng and Nowack [46] regarding the kinetic parameters and SF parameter using one compartment (OC) and one compartment with stored fraction (OC-SF) models for different aquatic invertebrates exposed to different NMs.

No.	Organism (Material, Exposure type)	Model	*C*_exposure_	*k*_1_	*k*_1_ (web-tool)	*k*_2_	*k*_2_ (web-tool)	SF	SF (web-tool)	Adj. R^2^	Adj. R^2^ (web-tool)	Ref.
1	*S. obliquus* (C_60_, aqueous)	OC	2	1697	1706.7	1.861	1.880	/	/	0.928	0.928	[15]
		OC-SF	2	2316	2245.9	2.713	2.631	0.002	0.002	0.953	0.950	
2	*D. magna* (TiO_2_, aqueous)	OC	1	14157	10069.1	0.420	0.265	/	/	0.924	0.935	[86]
		OC-SF	1	14330	10058.0	0.429	0.265	0.001	0.000	0.922	0.931	
3	*D. magna* (SiO_2_, aqueous)	OC	1	29596	27932.9	0.664	0.551	/	/	0.936	0.920	[86]
		OC-SF	1	31398	31057.1	0.729	0.650	0.003	0.005	0.940	0.932	
4	*D. magna* (C_60_, dietary)	OC	15.02	129	129.1	0.219	0.219	/	/	−0.107	−0.130	[15]
		OC-SF	15.02	348	359.1	0.832	0.872	0.014	0.014	0.783	0.780	
5	*D. magna* (graphene, aqueous)	OC	0.25	2729	2766.4	0.063	0.064	/	/	0.822	0.819	[16]
		OC-SF	0.25	4088	4078.8	0.162	0.162	0.125	0.125	0.858	0.855	
6	*D. magna* (GO, aqueous)	OC	5	2915	2959.1	0.177	0.119	/	/	0.972	0.973	[87]
		OC-SF	5	2830	2836.7	0.109	0.118	0.000	0.005	0.954	0.959	
7	*D. magna* (Au, aqueous)	OC	0.4	18897	13493.1	0.285	0.320	/	/	0.760	0.823	[17]
		OC-SF	0.4	18897	13546.7	0.285	0.323	0.000	0.001	0.820	0.798	
8	*D. rerio* (TiO_2_, aqueous)	OC	0.06	0.29	0.306	0.009	0.010	/	/	0.106	0.189	[88]
		OC-SF	0.06	0.62	0.711	0.033	0.039	0.058	0.051	0.345	0.450	

The robustness of the genetic algorithm in handling nonlinear complexities can be explained by the fact that unlike Gradient Descent, which can struggle with local minima, or Levenberg-Marquardt, which requires good initial parameter estimates and can be sensitive to the nature of the cost function, genetic algorithm operates with a population of solutions. [69], [71] This significantly enhances its ability to explore the parameter space more thoroughly and to avoid local optima therefore, making it particularly effective in navigating the complex, nonlinear landscapes that characterize the model parameter estimations of the two biokinetic models implemented here.

It is noteworthy that the configuration of the population size and the fitness limit in the genetic algorithm were derived through a comprehensive sensitivity analysis aimed at optimizing the balance between computational speed and the quality of the goodness-of-fit. Zheng and Nowack, [46] conducted an analysis of the uptake and bioaccumulation of nanomaterials in invertebrates based on data from 34 experiments. It has been observed that the elimination rate constant ($k_{2}$) typically ranges from 0.001 to 3.0. In our backend implementation, the range for $k_{2}$ has been hardcoded to span from 0.001 to 100.0 to expedite the computational fitting process and ensure convergence before reaching the limits set by population size or the fitness threshold. The upper limit for $k_{2}$ was set to 100.0 to account for a margin of error, as the 3.0 maximum reported by Zheng and Nowack [46] was based on a limited dataset of 34 studies. By extending the upper limit, potential variability is accommodated, allowing for greater flexibility in application. This adjustment allows the fitting process for all eight case studies, as detailed in Table 1 to be completed in a few seconds. This adjustment significantly reduced the processing time for executing the nonlinear fitting, from minutes to seconds yet ensures that the full range of potential values across NMs and species is covered. This highlights the tool’s enhanced ability to conduct rapid and robust nonlinear regression analyses efficiently. The current hardcoded range for $k_{2}$ is sufficiently broad to capture accurate fittings across a wide array of scenarios, regarding modelling the uptake and bioaccumulation of nanomaterials in aquatic and soil invertebrates.

### 3.2 Adjusted R-squared analysis

Once the kinetic parameters have been obtained, the statistical metric adjusted R-squared (*R*^2^) can be utilized in the *NanoBioAccumulate* web application to assess the goodness-of-fit of the nonlinear models corrected by the number of model parameters. The adjusted *R*^2^ is a statistical measure that quantifies the proportion of the variance in the dependent variable that is accounted for by the independent variable, considering any potential bias resulting from differences in the number of parameters between the two biokinetic models being compared. The adjusted *R*^2^ is calculated as (Eq. 5): [72], [73].

(5) $$R_{adj}^{2}=1-\frac{n-1}{n-p}\times \left(1-R^{2}\right)$$

where *n* is the sample size, *p* is the number of parameters, and *R*^2^ is the coefficient of determination calculated as (Eq. 6):

(6) $$R^{2}=1-\frac{RSS}{TSS}=1-\frac{\sum {\left(\gamma -\hat{\gamma }\right)}^{2}}{\sum {\left(\gamma -\bar{\gamma }\right)}^{2}}$$

where *RSS* is the residual sum-of-squares, *TSS* is the total sum-of-squares, *γ* is the response values, $\hat{\gamma }$ is the fitted values and $\overset{®}{\gamma }$ is the mean of response values.

### 3.3 Akaike Information Criterion (AIC) analysis

In addition to the adjusted R-squared, the Akaike Information Criterion (AIC) analysis is integrated in the *NanoBioAccumulate* web application to further assess the goodness-of-fit of the nonlinear models. The model with the lowest AIC value is considered to demonstrate the most favourable goodness-of-fit. The AIC is calculated as: [74], [75], [76].

(7) AIC=2p−2ln(L)

where *p* is the number of parameters in the model, and *L* is the maximum log-likelihood of the estimated model. In the case of a nonlinear fit with normally distributed errors, the ln(*L*) is calculated by:

(8) $$\ln\left(L\right)=0.5\times \left(-N\times \left(\ln\;2\pi +1-\;\ln\;N+\;\ln\;\sum_{i=1}^{n}x_{i}^{2}\right)\right)$$

However, in cases where the sample size is small and the ratio of sample (*n*) and number of parameters (*p*) is less than 40, a modification to the AIC is suggested. This modification, known as bias-corrected AIC (AICc) is calculated as follows: [76].

(9) $$AICc=AIC+\frac{2p\left(p+1\right)}{n-p-1}$$

where *n* is the sample size and *p* is the number of parameters.

Both the AIC and AICc are used for comparing the goodness-of-fit across different models; however, they do not serve as direct indicators of the quality of the fit itself. [77] To evaluate the validity of model fit, Akaike weights are utilized to quantify the weight of evidence for each model within a set of competing models. [77], [78] Akaike weights *w*_*i*_ (AIC) are calculated as follows: [77].

(10) $$w_{i}\left(AIC\right)=\frac{\exp\left\{-\frac{1}{2}\Delta_{i}\left(AIC\right)\right\}}{\sum_{k=1}^{K}\exp\left\{-\frac{1}{2}\Delta_{k}\left(AIC\right)\right\}}$$

where *i* and *k* denote the model numbers, while Δ_*i*_ (*AIC*) represents the difference in AIC between each model and the model with the lowest AIC. These differences are then normalized by dividing the sum of all such differences (denominator). In this context, the bias-corrected AICc was used to calculate the Akaike weights. An Akaike weight, such as *w*_*i*_ of 0.7 for a particular model, indicates that this model has 70 % probability of being the most appropriate choice among the models evaluated. The analysis of AIC, AICc and Akaike weights is integrated into the *NanoBioAccumulate* web application to support users in selecting the optimal model for their scenario.

## 4 The NanoBioAccumulate web application

The *NanoBioAccumulate* web application is a user-centric application developed with the ZK framework (an open-source Ajax Web application framework, written in Java), [79] and Java, which form an integral part of the Enalos Cloud platform (https://www.enaloscloud.novamechanics.com/). [80].

**Fig. 2:**
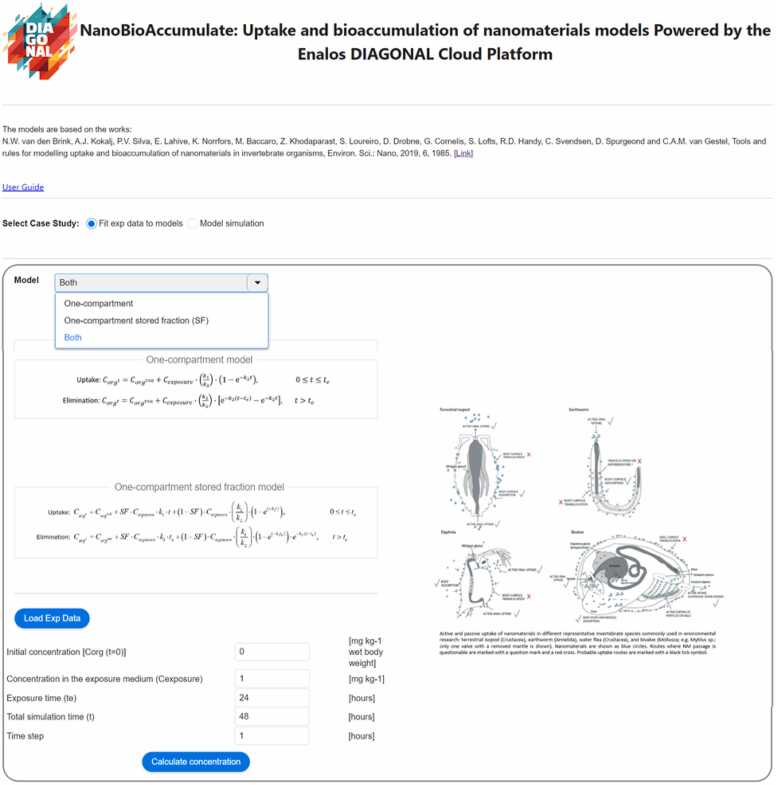
Display of the web interface of the NanoBioAccumulate tool for the case when kinetic parameters and SF parameter are unknown thus, the ‘Fit to exp data to models’ option is selected.

When the kinetic parameters and SF parameter are unknown, the *NanoBioAccumulate* web application offers the feature of performing nonlinear regression using a genetic algorithm. This allows to fit user-provided experimental data and estimate the unknown parameters of the two biokinetic models. The interface for this case is presented in Fig. 2 where the selected case study is the ‘Fit exp data to models’. The design of the interface is user-friendly, with only a few information input requirements. The user is prompted to select whether both biokinetic models will be utilized or if only one of the two models will be used. Additionally, the user is required to input values, including the initial concentration of the NM of interest in the organism, the concentration in the exposure medium, the exposure time, the total simulation time for both uptake and elimination (these parameters are sourced from previously reported experimental studies) and the time step for the analytical solution of the biokinetic models.

**Fig. 3:**
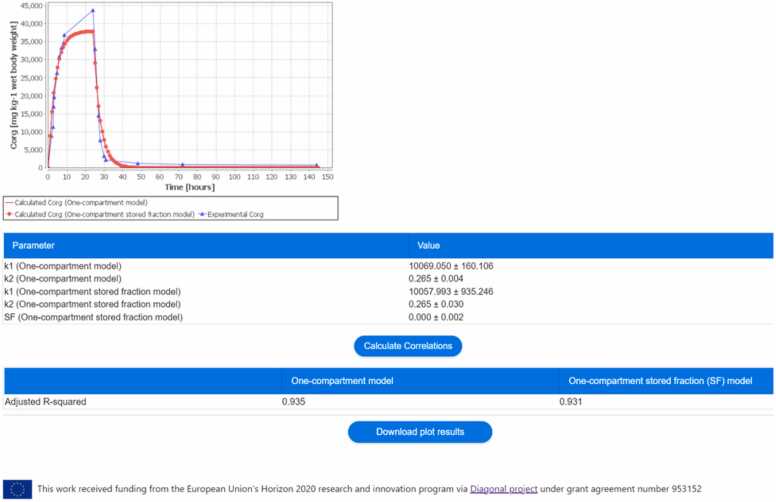
The NanoBioAccumulate web application generates a plot displaying the analytical solution from the two biokinetic models (if the ‘both’ models option is selected), along with the experimental data. Additionally, the web application generates two tables: one displaying the fitted values for the unknown parameters in the biokinetic models, and the other presenting the calculated adjusted R-squared values, providing an assessment of model fit for each of the models.

Upon clicking the ‘Calculate concentration’ button on the bottom page of the interface as shown in Fig. 2, a plot displaying the analytical solution from the two biokinetic models (if ‘both’ models option is selected), along with the experimental data, is generated, as shown in Fig. 3. Additionally, a Table is presented, containing the fitted values for the kinetic parameters *k*_1_ and *k*_2_ of the one-compartment (OC) model, as well as *k*_1_, *k*_2_, and the stored fraction (SF) of the one-compartment stored fraction (OC-SF) model. To further evaluate the goodness-of-fit corrected for the number of parameters for each model, the user can select the button ‘Calculate Correlations’ to estimate the adjusted R-squared value of each model. Furthermore, users have the option to download the plot results by selecting the ‘Download plot results’ button, enabling them to access the analytical solution of the two biokinetic models.

**Fig. 4:**
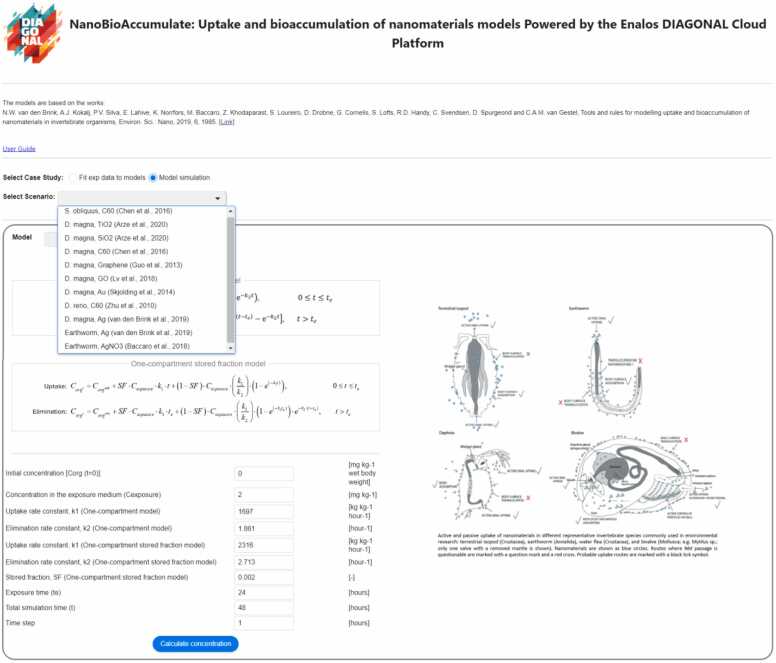
Display of the web interface of the NanoBioAccumulate tool for the case when kinetic parameters and SF parameter are unknown or the user would like to select among predefined scenarios thus, the ‘Model scenario’ is selected.

When the kinetic parameters and the SF are already known, or the user wants to explore different pre-defined model scenarios regarding the uptake and elimination kinetics of various aquatic and soil invertebrates (exposed to different NMs) the ‘Model simulation’ feature of the interface is selected. As illustrated in Fig. 4, this feature allows the user to select from a range of pre-defined scenarios (test organism and NM to which the organisms were exposed), each of which will automatically update the input fields based on the chosen scenario. The user has also the flexibility to manually input the various parameters for the models. These parameters include the initial concentration, concentration in the exposure medium, *k*_1_ and *k*_2_ for OC model and *k*_1_, *k*_2_ and SF for OC-SF model, exposure time, total simulation time for both uptake and elimination (sourced from previously reported experimental or analytical studies) and the time step for analytical solution of the biokinetic models.

**Fig. 5:**
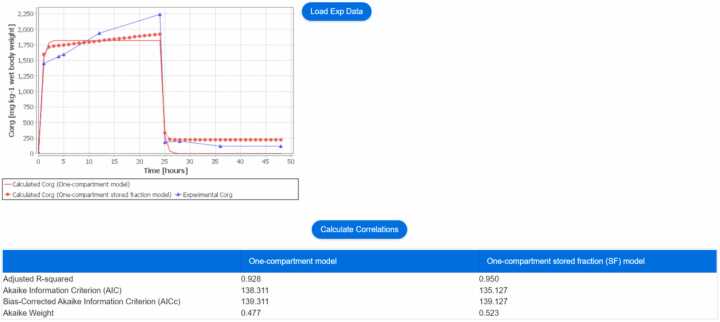
The NanoBioAccumulate web application generates a plot displaying the analytical solution from the two biokinetic models (if the ‘both’ models option is selected), along with the experimental data when ‘Model simulation’ is selected as a case study. It also generates a table with the calculated adjusted R-squared values for each model.

After clicking the ‘Calculate concentration’ button on the bottom page of the interface as shown in Fig. 4, a plot is generated showcasing the analytical solution derived from the two biokinetic models, when ‘both’ models option is selected. Additionally, users can upload experimental data by clicking on the ‘Load Exp Data’ button (Fig. 5). This experimental data is consequently presented on the plot. The loaded experimental data can then be used to calculate the adjusted R-squared value, allowing for an evaluation of the goodness-of-fit for each biokinetic model.

**Fig. 6:**
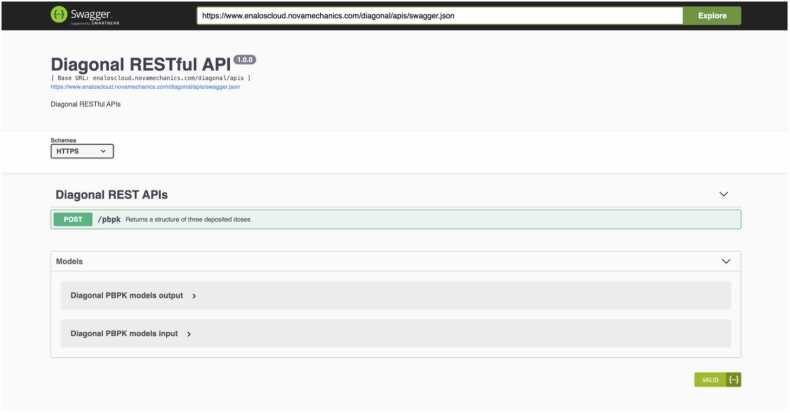
NanoBioAccumulate is available through a REST API to enable programmatic access and integration with other models, tools and into an IATA for hazard and risk assessment.

To facilitate communication with other services (e.g., databases, other models, or as part of an IATA) and web applications, *NanoBioAccumulate* incorporates a Representational State Transfer (REST) application programming interface (API) (see Fig. 6), enhancing its interoperability and functionality within the broader scientific and environmental research community. The *NanoBioAccumulate* web application effectively reduces the barriers for conducting intricate scientific computations by eliminating the need for programming skills. Its integration into the Enalos Cloud Platform further enhances its capabilities by enabling users to access and utilize various other web applications hosted on the same platform. [56], [81], [82], [83], [84] This integration is particularly advantageous for users interested in SSbD approaches, as it expands user accessibility to a wide range of tools that advance accessibility and enhance overall capabilities.

*NanoBioAccumulate’s* API can facilitate numerous practical applications, one of which includes integration with environmental monitoring databases such as the US EPA’s CompTox Chemicals Dashboard. [85] Through this integration, users can fetch real-time data on nanomaterials’ environmental concentration in soil or aquatic environments and utilize this real-time information within the integrated biokinetic models of *NanoBioAccumulate* to model the uptake and bioaccumulation of nanomaterials within soil or aquatic invertebrates.

Additionally, the API can facilitate integration with Laboratory Information Management Systems (LIMS) in toxicological research laboratories. This integration can enable the automatic extraction of metadata, including information related to nanomaterial’s uptake and bioaccumulation in invertebrates and experimental conditions. These extracted data can be utilized by *NanoBioAccumulate* to calibrate the biokinetic models, by calculating the uptake and elimination rate constants as well as the stored fraction constant, through the application of the nonlinear regression featured in this web-based tool.

Moreover, the API’s capabilities can extend to foster collaborative projects and enhance data transparency through integration with cloud-based platforms such as Google Cloud or Amazon Web Services. This aspect is particularly beneficial for disseminating research findings and supporting the development of regulatory policies and guidelines.

In summary, the API capabilities of *NanoBioAccumulate* can advance ecotoxicological research by facilitating the optimization of nanomaterial concentrations and compositions that effectively mitigate adverse effects in invertebrates. This pivotal role in guiding the development of safer nanomaterials holds significant promise in reducing ecological and health risks.

## 5 Case studies

To verify the performance and accuracy of the *NanoBioAccumulate* web application and demonstrate its ability to accurately quantify kinetic parameters and SF parameter for modelling the uptake and elimination of NMs in soil and aquatic invertebrate organisms, several case studies are presented below.

### 5.1 Kinetics of silver (Ag) accumulation in *D. magna* exposed for 48 h to Ag-NP through water

**Fig. 7:**
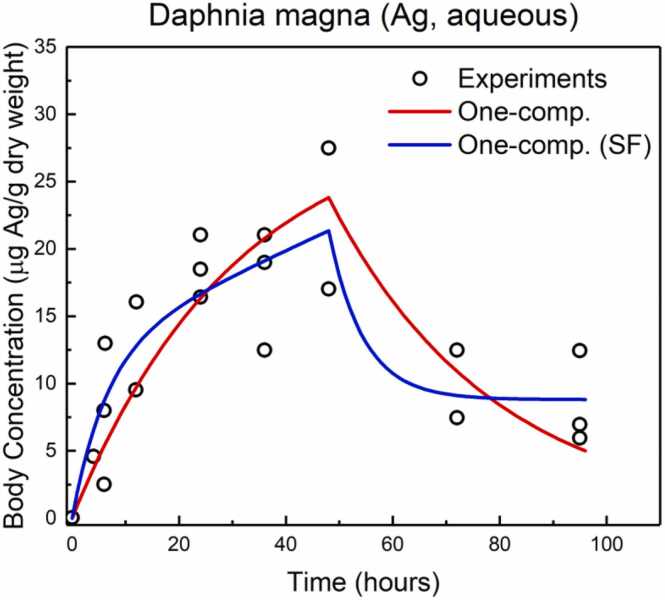
NanoBioAccumulate web application output with experimental data [18] for the one compartment model and one compartment with a stored fraction (SF) model for modelling the kinetics of Ag in D. magna exposed for 48 h to Ag-NP through water.

Ribeiro et al. [18] conducted experiments to calculate the kinetics of silver (Ag) accumulation in *D. magna* exposed to Ag-NP through water for 48 h. The experimental results reported by Ribeiro et al. [18] are presented as open circles in Fig. 7. To calibrate the biokinetic models implemented in the *NanoBioAccumulate* web application, the experimental dataset provided by Ribeiro et al. [18] was used. This dataset was employed to estimate the kinetic parameters and the stored fraction (SF) parameter through the nonlinear regression fitting provided within the *NanoBioAccumulate* web application.

For the OC model, the estimated values from *NanoBioAccumulate* for *k*_1_ (L_medium_ kg_organism_^−1^ hour^−1^) and *k*_2_ (hour^−1^) were equal to 0.196 and 0.033, respectively. For the OC-SF model, the estimated values for *k*_1_, *k*_2_, and SF were equal to 0.426, 0.155 and 0.086, respectively. The calculated adjusted R squared for OC model was found to be 0.594 and for the OC-SF model was found to be 0.690. To evaluate the validity of the model fit, the Akaike weights were calculated, resulting in a value of 85.7 % for the OC-SF model. This suggests that the OC-SF model has an 85.7 % probability of being the most appropriate choice in comparison to the OC model. The accumulated concentration of Ag in the body of *D. magna* as calculated (by using the proposed fitted values) from the *NanoBioAccumulate* is presented in Fig. 7 using the OC model (red line) and the OC-SF model (blue line), showing good correlation with the experimental data points.

Van den Brink et al., [37] employed nonlinear regression analysis using Genstat [65] to quantify the kinetic parameters and SF by using the experimental data from Ribeiro et al. [18] and found that for the OC model, the estimated values were *k*_1_ = 0.1897 ± 0.025 and *k*_2_ = 0.03107 ± 0.00654. For the OC-SF model, the estimated values were *k*_1_ = 0.363 ± 0.121, *k*_2_ = 0.124 ± 0.0587 and SF = 0.0991 ± 0.0327. The calculated adjusted R-squared for the OC model was found to be 0.595 and for the OC-SF model was 0.695. The *NanoBioAccumulate* web application shows very good agreement (within the margin of statistical errors) with the findings of Van den Brink et al. [37] This agreement provides a strong basis for affirming the reliability and accuracy of the *NanoBioAccumulate* web application in accurately predicting the kinetics of silver (Ag) accumulation in *D. magna* through water uptake.

### 5.2 Kinetics of accumulation of total Ag in earthworm tissue exposed to Ag-NPs and AgNO_3_

**Fig. 8:**
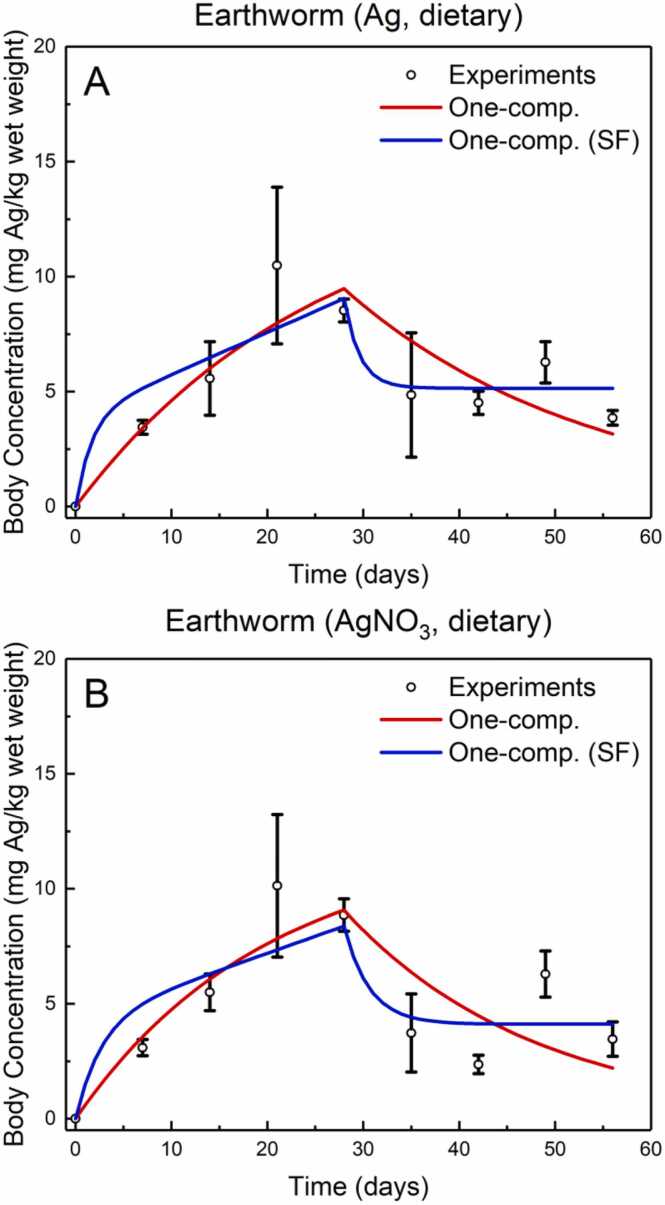
NanoBioAccumulate web application output with experimental data [19] for the one compartment (OC) model and the one compartment with a stored fraction (OC-SF) model for modelling the kinetics of Ag accumulation in earthworms exposed to (a) Ag-NPs and (b) AgNO_3_ (error bars represent standard deviation calculated by Baccaro et al. [19] from averaging 4 experimental replicates).

Baccaro et al. [19] conducted experiments to calculate the kinetics of silver (Ag) accumulation in earthworm tissue when exposed to Ag-NPs and AgNO_3_, respectively. To further validate the *NanoBioAccumulate* web application, the experimental dataset provided by Baccaro et al. [19] was used, shown as open circles in Figs. 8A and 8B. Utilizing the *NanoBioAccumulate* web application, the kinetic parameters and SF parameter were quantified to compare and validate the results against the experimental findings obtained by Baccaro et al. [19]. As in the previous case study example, the nonlinear regression feature of the web application is utilized to quantify these parameters.

The kinetics of Ag accumulation in earthworms exposed to Ag-NPs were initially calculated. The OC model was first employed, and the kinetic rate constants *k*_1_ (kg per kg per day) and *k*_2_ (per day) determined using the *NanoBioAccumulate* web-tool were found to be equal to 0.060 and 0.039, respectively. The adjusted R-squared was computed to be 0.653. This is in excellent agreement with the kinetics proposed in the work of Baccaro et al. [19] where *k*_1_ was reported as 0.061 ± 0.019 and *k*_2_ as 0.040 ± 0.050 thus, demonstrating the high accuracy and reliability of the web application's predictions.

Furthermore, the web application provides users with the capability to utilize the OC-SF model to quantify the kinetic parameters and expand further the analysis. The obtained values for *k*_1_, *k*_2_, and SF were 0.278, 0.616, and 0.071, respectively, with an adjusted R-squared value of 0.675. This illustrates that the OC-SF model performs slightly better in terms of goodness-of-fit compared to the OC model for this specific example. However, when assessing model fit using Akaike weights, the OC model is found to outperform the OC-SF model in this case, with 65.2 % probability of being the most appropriate choice. Despite the OC model having a slightly lower adjusted R-squared value than the OC-SF model, this outcome highlights the importance of considering multiple criteria when determining the best-fitting model. Overall, this demonstrates the capability of the *NanoBioAccumulate* web application to guide users in selecting the most appropriate model that fits well to experimental data for further analysis. The fits for both the OC model and the OC-SF model are shown in Fig. 8A.

The kinetics of accumulation of total Ag in earthworms exposed to AgNO_3_ are explored next. Fig. 8B, presents the results as obtained from the two biokinetic models integrated in the *NanoBioAccumulate* web application and demonstrates its comparison against the experimental data obtained by Baccaro et al. [19] The *NanoBioAccumulate* web-tool predicted for the OC model, generated values of *k*_1_ 0.062 and *k*_2_ 0.049. The values reported by Baccaro et al. [19] for *k*_1_ were 0.055 ± 0.007 and for *k*_2_ were 0.040 ± 0.018. The adjusted R-squared obtained using the proposed kinetics from the *NanoBioAccumulate* was 0.536, which is slightly higher than the value of 0.491 obtained based on the kinetics proposed by Baccaro et al. [19]. This highlights the slightly better performance of *NanoBioAccumulate* in accurately predicting kinetics through effectively fitting to the experimental data.

The analysis is further extended using the *NanoBioAccumulate* web application to estimate the kinetic parameters using the OC-SF model. The calculated value for *k*_1_ was 0.196, for *k*_2_ was 0.379 and for SF was 0.083. The adjusted R-squared calculated was 0.587 thus, suggesting that the utilization of the OC-SF model gives a slightly improved model fit compared to the conventional OC model. However, when evaluating model fit through Akaike weights, the OC model is found to outperform with a 65.5 % probability of being the best-fitting model. This underscores the need to consider multiple metrics, rather than relying solely on adjusted R-squared, when determining which model offers the best goodness-of-fit. Overall, the findings demonstrate that the models calibrated using the *D. magna* dataset (as described in Section 6.1) are applicable to other invertebrate species and nanomaterials, thereby underscoring the robustness and generalizability of these models.

### 5.3 Domain of applicability and user interaction with the model

To assess the broad applicability of the *NanoBioAccumulate* web-tool and its user interaction capabilities, a comprehensive analysis with various examples was conducted to explore the various domains where the tool could be utilized and discuss the ways in which users can interact with the integrated biokinetic models.

Zheng and Nowack [46] collected 34 datasets that provide insights into the uptake and elimination kinetics of insoluble NMs in aquatic invertebrate organisms, including *Scenedesmus obliquus* (green algae), *Daphnia magna* (water flea) and Danio rerio (zebrafish). In this work, 8 datasets were adopted from the work of Zheng and Nowack [46] as shown in Table 1, to assess the suitability and effectiveness of the *NanoBioAccumulate* web application in estimating the uptake and elimination kinetic parameters of various poorly soluble or insoluble NMs. Specifically, these datasets involved the examination of *S. obliquus* exposed to fullerene-C_60_ NMs, [15] *D. magna* exposed to titanium dioxide (TiO_2_), silicon dioxide (SiO_2_), fullerene-C_60_, graphene, graphene oxide (GO), gold (Au) NMs [15], [16], [17], [86], [87] and *D. rerio* exposed to TiO_2_. [88].

According to Van den Brink et al. [37] and Zheng and Nowack [46] the majority of uptake experiments involving NMs are conducted using total concentrations. This is because it remains difficult to accurately track the conversion between NMs and soluble ions, as well as being challenging to distinguish the accumulation of NMs and ions within organisms. Hence, many studies in the literature focus on investigating insoluble NMs, where only the uptake of particulate matter is considered, rather than the uptake of dissolved metals resulting from the partial dissolution of NMs. The examples presented in Table 1 (obtained from the study of Zheng and Nowack [46]) exclusively focus on non-soluble NMs and adult aquatic invertebrates.

Table 1 provides a detailed comparison between the estimated uptake and elimination kinetics parameters for the different aquatic invertebrates exposed to different NMs using the *NanoBioAccumulate* web tool and the results obtained by Zheng and Nowack [46] The latter authors used the GraphPad Prism version 9.3.1 [60] to perform nonlinear regression fitting on experimental data to quantify the kinetic parameters and SF parameter. Additionally, the calculated adjusted R-squared values are compared to measure the quality of fit for each proposed kinetic parameters and model. In each example the $C_{org^{t=0}}$ was set at zero. The concentration in the exposure medium (*C*_exposure_) is measured in units of mg L^−1^ where the uptake rate constant *k*_1_ has units of L kg^−1^ h^−1^ and the elimination rate constant *k*_2_ has units h^−1^.

The results presented in Table 1 consistently demonstrated that the *NanoBioAccumulate* web tool aligns very well with the estimated kinetic parameters and SF parameter calculated in the study by Zheng and Nowack [46] The Supporting Information file includes graphical plots for each case study outlined in Table 1. These plots demonstrate the correlation between the reported experimental data and the predictions generated by the *NanoBioAccumulate* tool for the OC and OC-SF models. Any slight discrepancies observed in Table 1 between the results obtained from *NanoBioAccumulate* and the values reported by Zheng and Nowack [46] can be likely attributed to potential inconsistencies in the digitization process of the experimental results. This is particularly apparent in Table 1 for case study 2, case study 7 and case study 8, where Figs. S2, S7 and S8 present inconsistent trends in the reported experimental values. Moreover, the study by Zheng and Nowack [46] does not clearly specify whether all experimental data points were included or if any outliers were excluded during the nonlinear fitting procedure. Nevertheless, very good agreement is consistently observed in the calculated adjusted R-squared values for each case examined.

Hence, it is safe to conclude that the *NanoBioAccumulate* web tool possesses the versatility, accuracy, and reliability necessary to model the uptake and elimination kinetics of NMs in a diverse range of aquatic and soil invertebrates. Furthermore, the web tool also integrates these specific model scenarios as predefined options, allowing users to gain insights from previous studies and the associated computed kinetics values. Consequently, this enhances user's understanding of NMs uptake and elimination in different organisms. Moreover, the web tool offers the flexibility to include additional model scenarios if required.

## 6 Conclusions

In this work, the *NanoBioAccumulate*, a web-tool is presented for the prediction of NMs uptake and elimination kinetics in organisms. *NanoBioAccumulate* is a free-to-use online web tool hosted on the Enalos DIAGONAL Cloud Platform (https://www.enaloscloud.novamechanics.com/diagonal/pbpk/) that empowers researchers to make informed decisions regarding the potential hazards and risks associated with the accumulation of NMs in aquatic and soil invertebrates. It is designed to offer a user-friendly interface, making it accessible to researchers from diverse backgrounds, without the need for programming skills.

The *NanoBioAccumulate* web application integrates two commonly used biokinetic models to simulate the uptake and elimination kinetics of NMs in organisms; the OC model and the OC-SF model. Notably, this application features an advanced genetic algorithm allowing the execution of nonlinear regression fitting on user-provided experimental data to compute the kinetics and SF parameter if these are unknown, thus bypassing the need for use of commercial statistical analysis software for calculating these parameters. While alternative software packages, such as R, exist for this purpose, the advantage of the *NanoBioAccumulate* lies in its user-friendly interface, making it suitable for individuals lacking software expertise. Furthermore, as a web-based tool, it eliminates the need for local compilation or installation, enabling users to access it effortlessly from any location. The high efficacy of the nonlinear regression provided in the *NanoBioAccumulate* web application is demonstrated by the various case studies presented in this study. [15], [16], [17], [18], [19], [37], [46], [86], [87], [89] The web application also provides various statistical metrics, including the calculation of adjusted R-squared and the bias-corrected Akaike Information Criterion (AICc), for assessing goodness-of-fit. These tools emphasize the importance of considering multiple metrics when determining the best-fitting model. For instance, although the OC-SF model demonstrated a slightly better adjusted R-squared in some cases, the Akaike weights revealed that the OC model outperformed the OC-SF model in terms of fitting accuracy in the earthworm case, while the reverse was true in the daphnia accumulation of AgNPs. This highlights the need to look beyond a single metric, such as adjusted R-squared, when evaluating model performance.

The web application demonstrated a broad range of applicability and enhanced user interaction by incorporating multiple predefined model scenarios. These scenarios have been precalculated using data from previous studies, focusing on the kinetics of uptake and elimination of various NMs such as TiO_2_, SiO_2_, C_60_, graphene, GO, Au, Ag, and AgNO_3_ in aquatic and soil invertebrates. This feature easily allows users to explore and analyse the behavior of these NMs in different organisms without the need to generate new kinetic models for each scenario.

*NanoBioAccumulate* provides insights into the uptake and elimination rates of various NMs-species, with access also available programmatically via an extensive API. This API allows for integration with other software and web applications, enabling efficient data exchange and interoperability. This advanced integration enables the transfer of data between *NanoBioAccumulate*, opening new possibilities for collaborative research and comprehensive environmental risk assessment. It also facilitates the integration of *NanoBioAccumulate* into existing environmental surveillance and monitoring systems, enhancing their capability to accurately track and assess the impact of NMs in the environment. This application will thus play a crucial role in enhancing our understanding of NMs’ impacts on the environment and human health, ultimately contributing to the responsible development and use of nanotechnology.

Future work to further enhance the functionality and applicability of this web application includes integration of additional biokinetic models. For instance, models that will be incorporated into the next iteration include the Michaelis-Menten equation, a commonly used approach in enzyme kinetic studies, [90] as well as biokinetic models that account only for the stored fraction during elimination and not during the uptake. Also, a biokinetic model that considers the incorporation of a growth dilution term in the one compartment model could be integrated into the web application. Numerous studies have reported that the increase in an organism’s mass over the course of an experiment can potentially result in a decrease in the internal concentration of substance in growing organisms. [91] Lastly, the inclusion of a model that can monitor the uptake and elimination of both total particulates and also of metal ions could be considered being integrated in the future. This example is apparent; when considering transformation and ageing of highly soluble NMs, specifically metal based NMs like Ag NMs which are prone to releasing Ag^+^ ions into the environment. [37] In addition to these models, the importance of incorporating an adsorption mechanism in future models to be integrated into the web application is acknowledged, as this is an important potential uptake mechanism as highlighted by Fig. 1. Including such a mechanism would offer a more comprehensive understanding of the processes involved in biokinetic modelling and improve the predictive accuracy of the application.

## Supporting information

A supporting information file is included, providing a comprehensive analysis that compares the results obtained from the *NanoBioAccumulate* web-tool with the experimental results reported in previous studies.

## Funding

This research was funded by the European Union's Horizon 2020 research and innovation program via Diagonal project under grant agreement number 953152).

## CRediT authorship contribution statement

Conceptualization, D.M, N.C; Methodology, D.M, N.C., N.B.; Software, D.M, N.C., A.T.; Supervision, N.B, H.L., G.M, I.L, A.A.; Writing—original draft preparation, D.M.; Writing—review and editing, D.M., N.C., A.T., A.P., N.B., H.L., G.M., I.L., A.A; Funding acquisition, N.B., H.L, I.L., A.A.

## Conflict of Interest

DGM, NC, AT, AGP, AA are affiliated with NovaMechanics, a cheminformatics and materials informatics company.
