# Supplementary material for: NanoBioAccumulate: Modelling the uptake and bioaccumulation of nanomaterials in soil and aquatic invertebrates via the Enalos DIAGONAL Cloud Platform
Source: Comput Struct Biotechnol J. 2024 Oct 17;25:243–55. doi: 10.1016/j.csbj.2024.09.028 (PMC11550214; doi:10.1016/j.csbj.2024.09.028)
Supplement: Supplementary file 1 — Supplementary material [file mmc1.docx]

Supplementary Materials for “*NanoBioAccumulate*: Modelling the uptake and bioaccumulation of nanomaterials in soil and aquatic invertebrates via the Enalos DIAGONAL Cloud Platform”

Dimitris G. Mintis,^a, b^ Nikolaos Cheimarios,^a, b^ Andreas Tsoumanis,^a, b^ Anastasios G. Papadiamantis,^a, b, c^ Nico W. van den Brink,^d^ Henk J. van Lingen,^e^ Georgia Melagraki,^f^ Iseult Lynch,^b, c^  Antreas Afantitis^a, b, g^

^a^ NovaMechanics Ltd., Nicosia 1070, Cyprus

^b^ Entelos Institute, Larnaca 6059, Cyprus

^c^ School of Geography, Earth and Environmental Sciences, University of Birmingham, Birmingham B15 2TT, United Kingdom

^d^ Department of Toxicology, Wageningen University, Wageningen, The Netherland

^e^ Laboratory of Systems and Synthetic Biology, Wageningen University & Research, Wageningen, the Netherland

^f^ Division of Physical Sciences and Applications, Hellenic Military Academy, Vari 16672, Greece

^g^ NovaMechanics MIKE, Piraeus 18545, Greece

# *NanoBioAccumulate* results compared to experiments


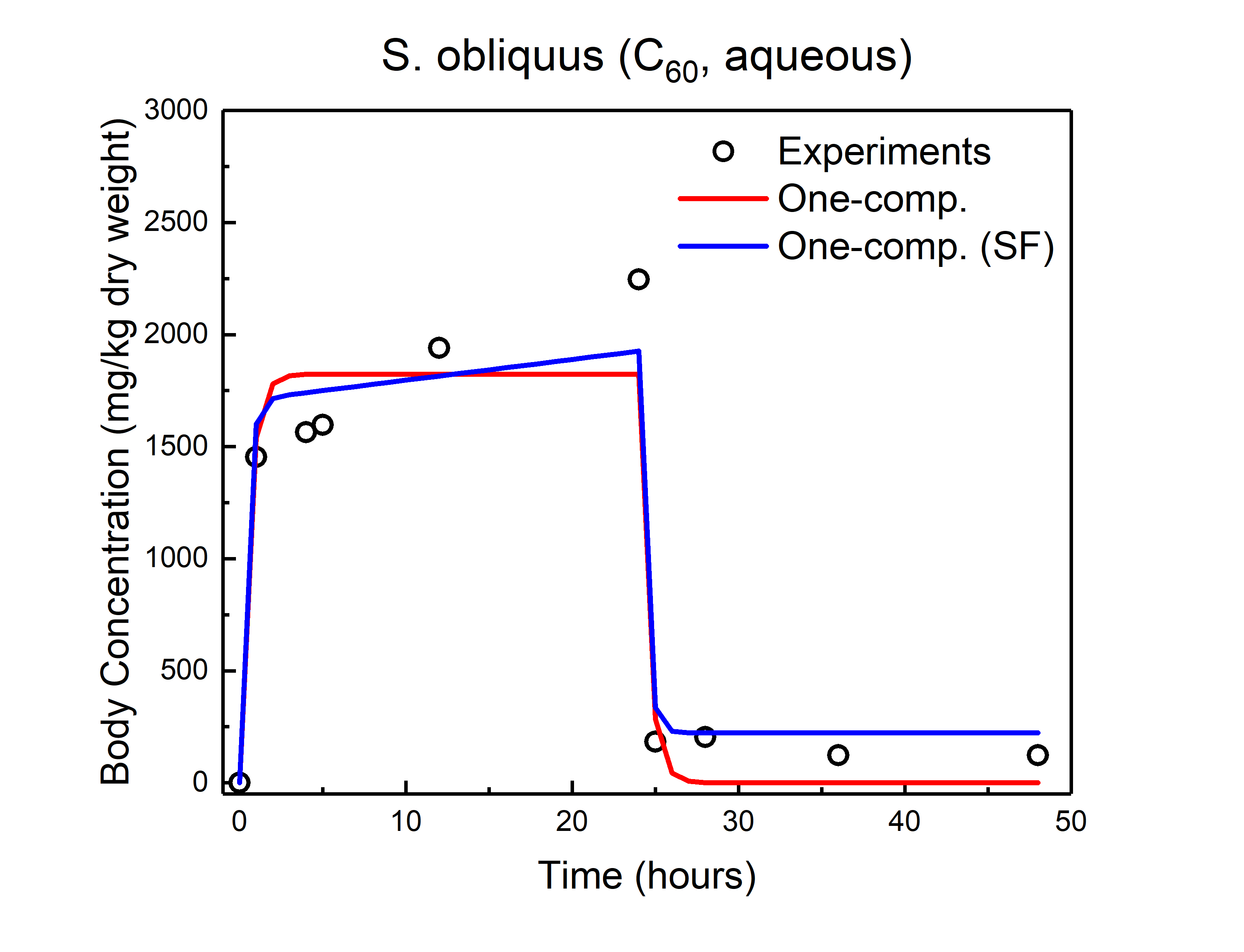


**Figure S1.** NanoBioAccumulate web application output with experimental data^1^ for the one compartment model and one compartment with a stored fraction (SF) model for modeling the kinetics of fullerene-C_60_ in S. obliquus exposed in aqueous environment.


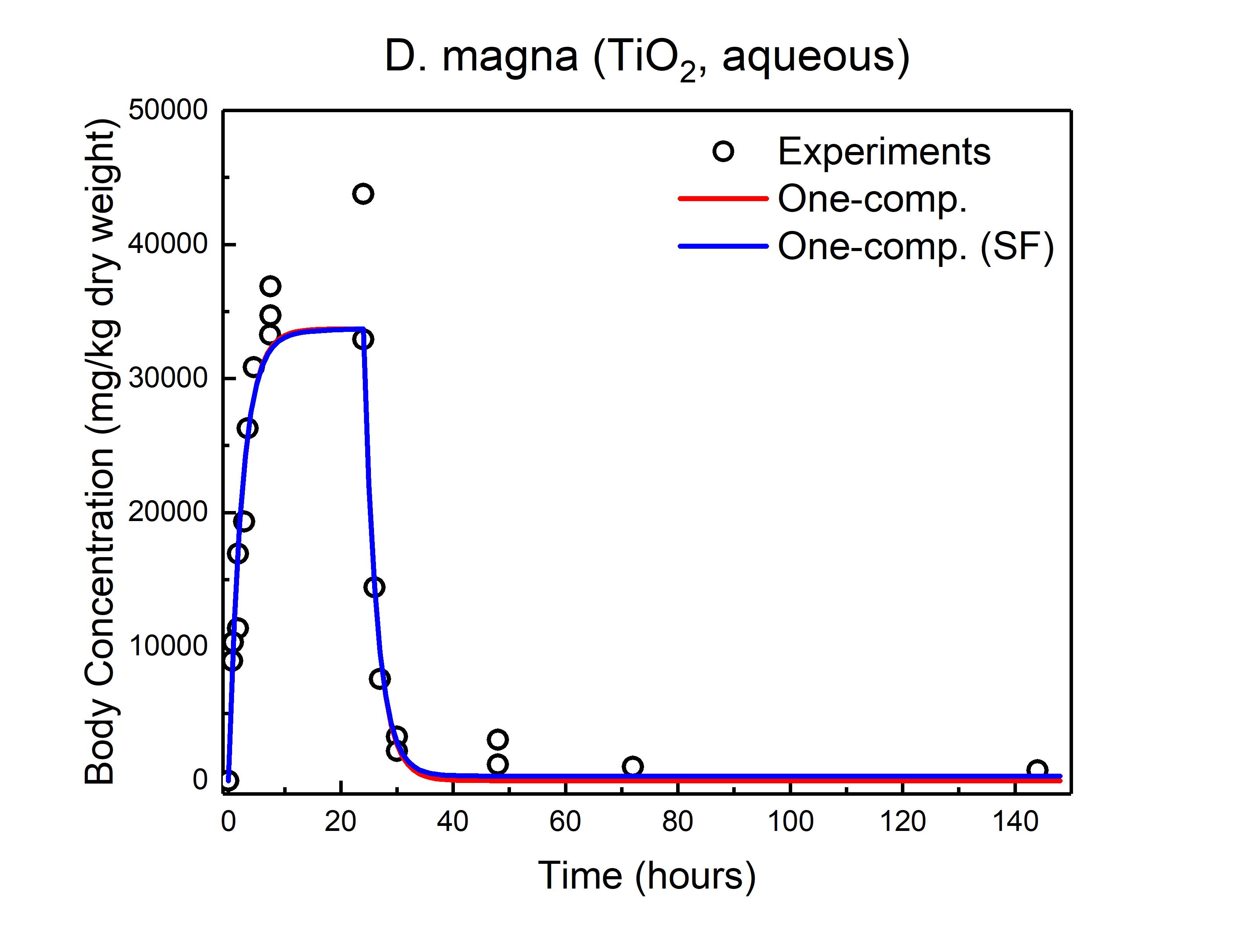


**Figure S2.** NanoBioAccumulate web application output with experimental data^2^ for the one compartment model and one compartment with a stored fraction (SF) model for modeling the kinetics of TiO_2_ in D. magna exposed in aqueous environment.


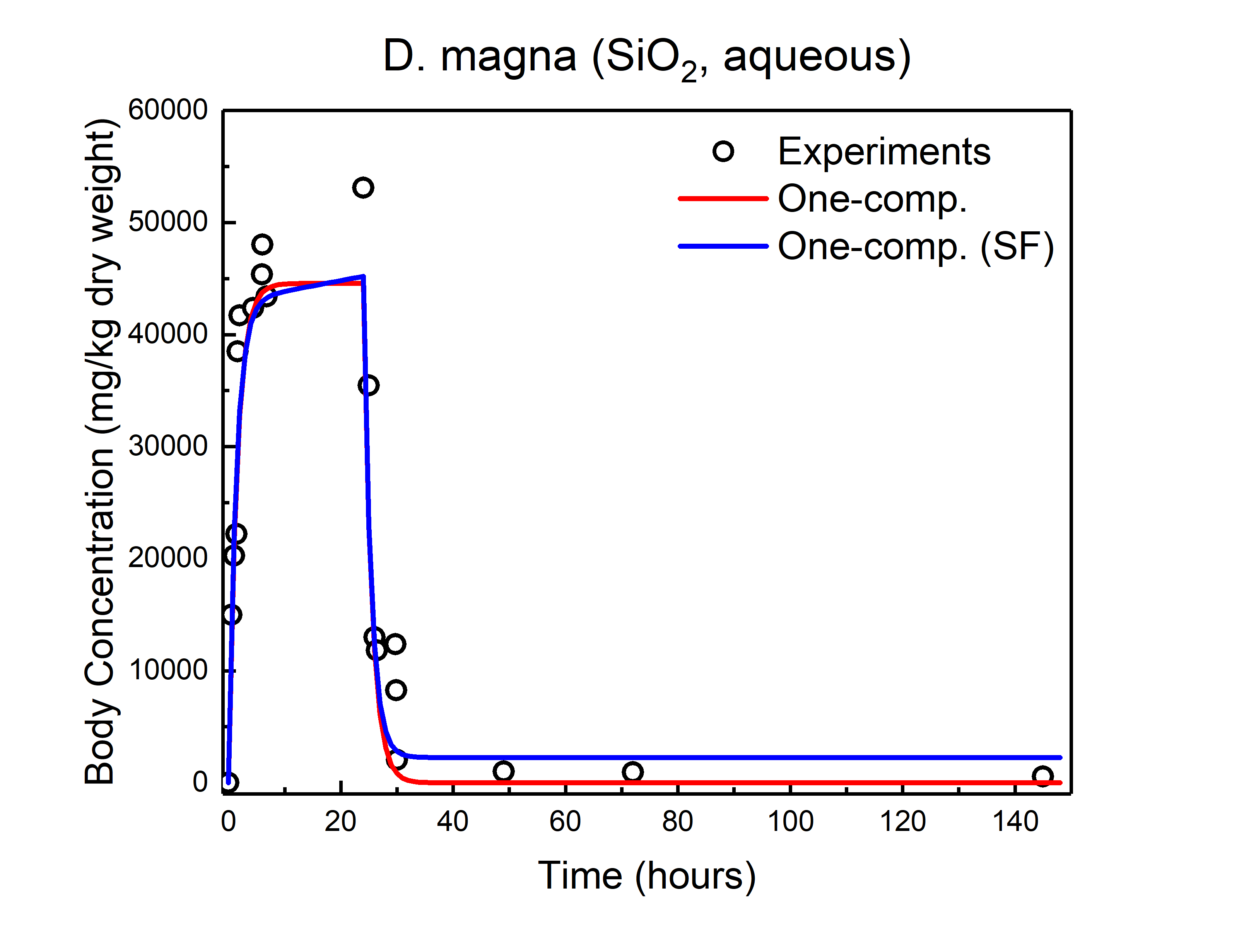


**Figure S3.** NanoBioAccumulate web application output with experimental data^2^ for the one compartment model and one compartment with a stored fraction (SF) model for modeling the kinetics of SiO_2_ in D. magna exposed in aqueous environment.


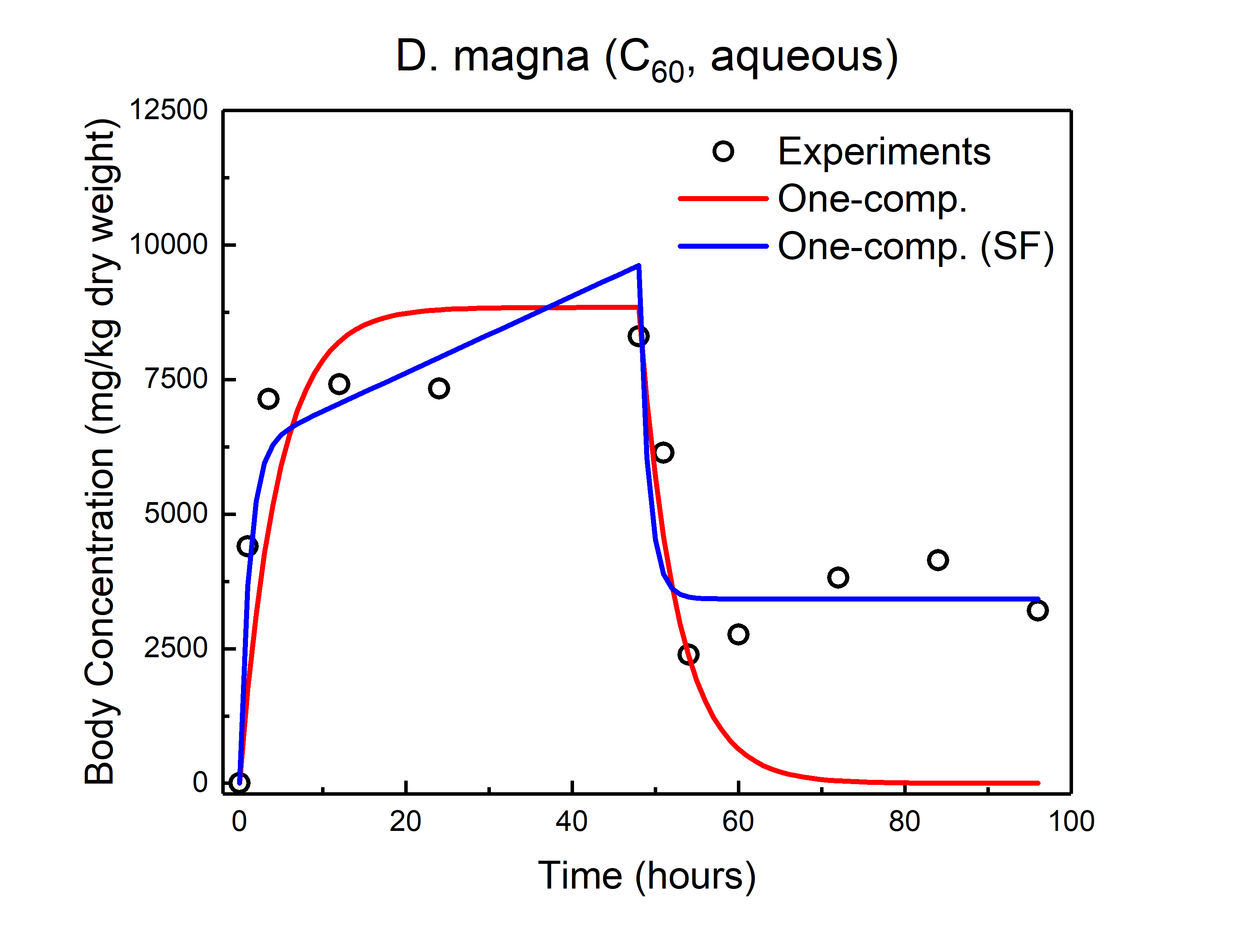


**Figure S4.** NanoBioAccumulate web application output with experimental data^1^ for the one compartment model and one compartment with a stored fraction (SF) model for modeling the kinetics of fullerene-C_60_ in D. magna exposed in aqueous environment.


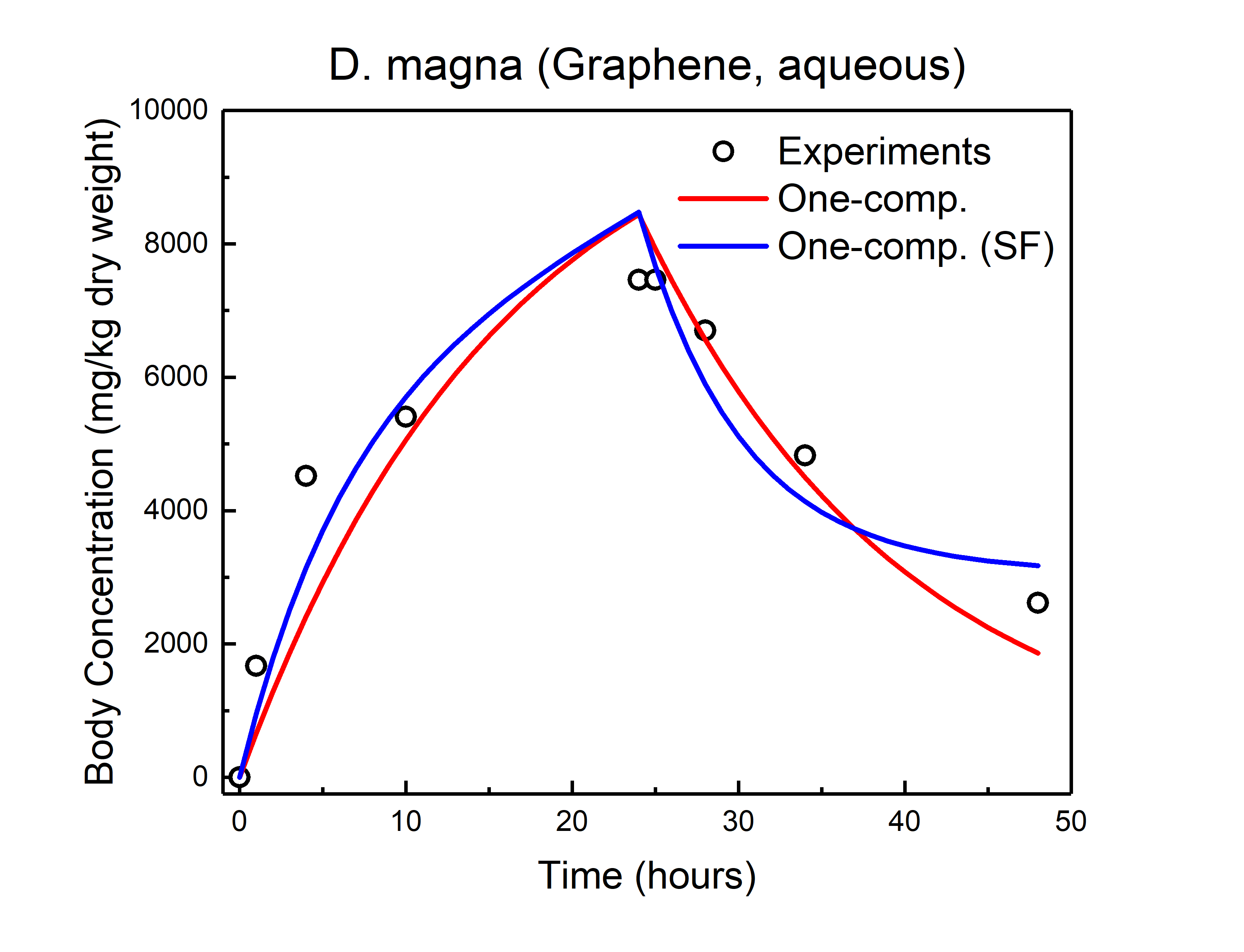


**Figure S5.** NanoBioAccumulate web application output with experimental data^3^ for the one compartment model and one compartment with a stored fraction (SF) model for modeling the kinetics of graphene in D. magna exposed in aqueous environment.


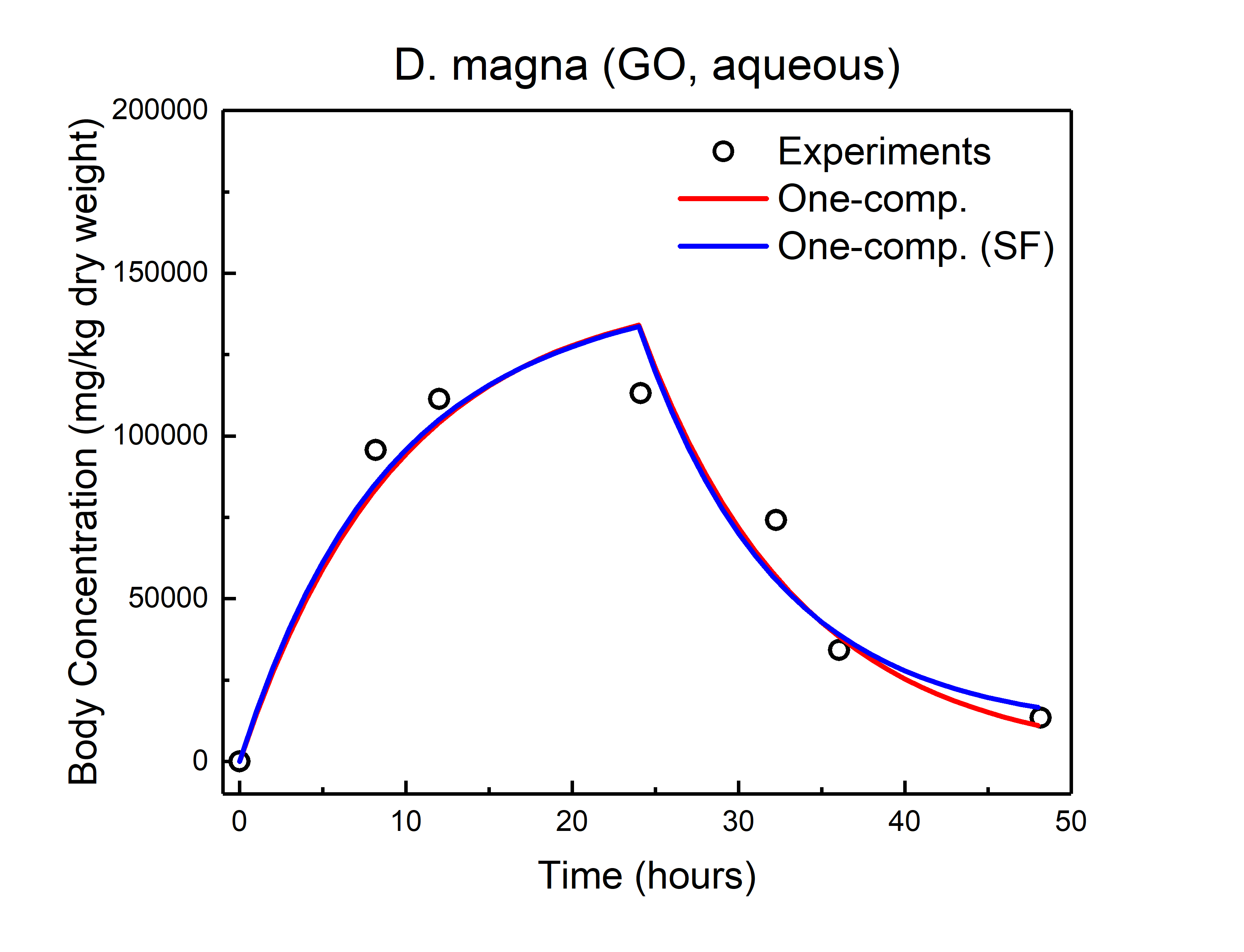


**Figure S6.** NanoBioAccumulate web application output with experimental data^4^ for the one compartment model and one compartment with a stored fraction (SF) model for modeling the kinetics of graphene oxide in D. magna exposed in aqueous environment.


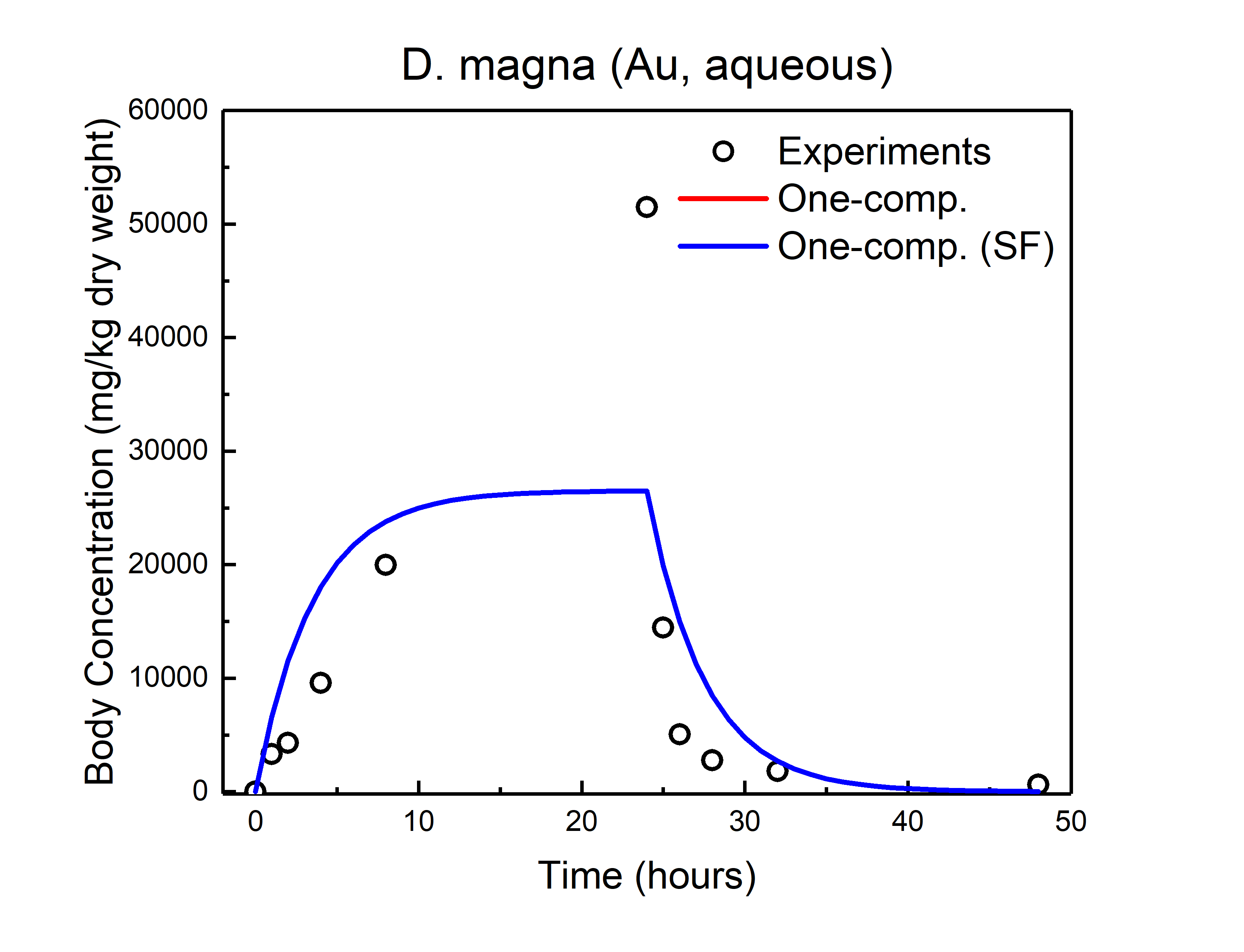


**Figure S7.** NanoBioAccumulate web application output with experimental data^5^ for the one compartment model and one compartment with a stored fraction (SF) model for modeling the kinetics of gold (Au) in D. magna exposed in aqueous environment.


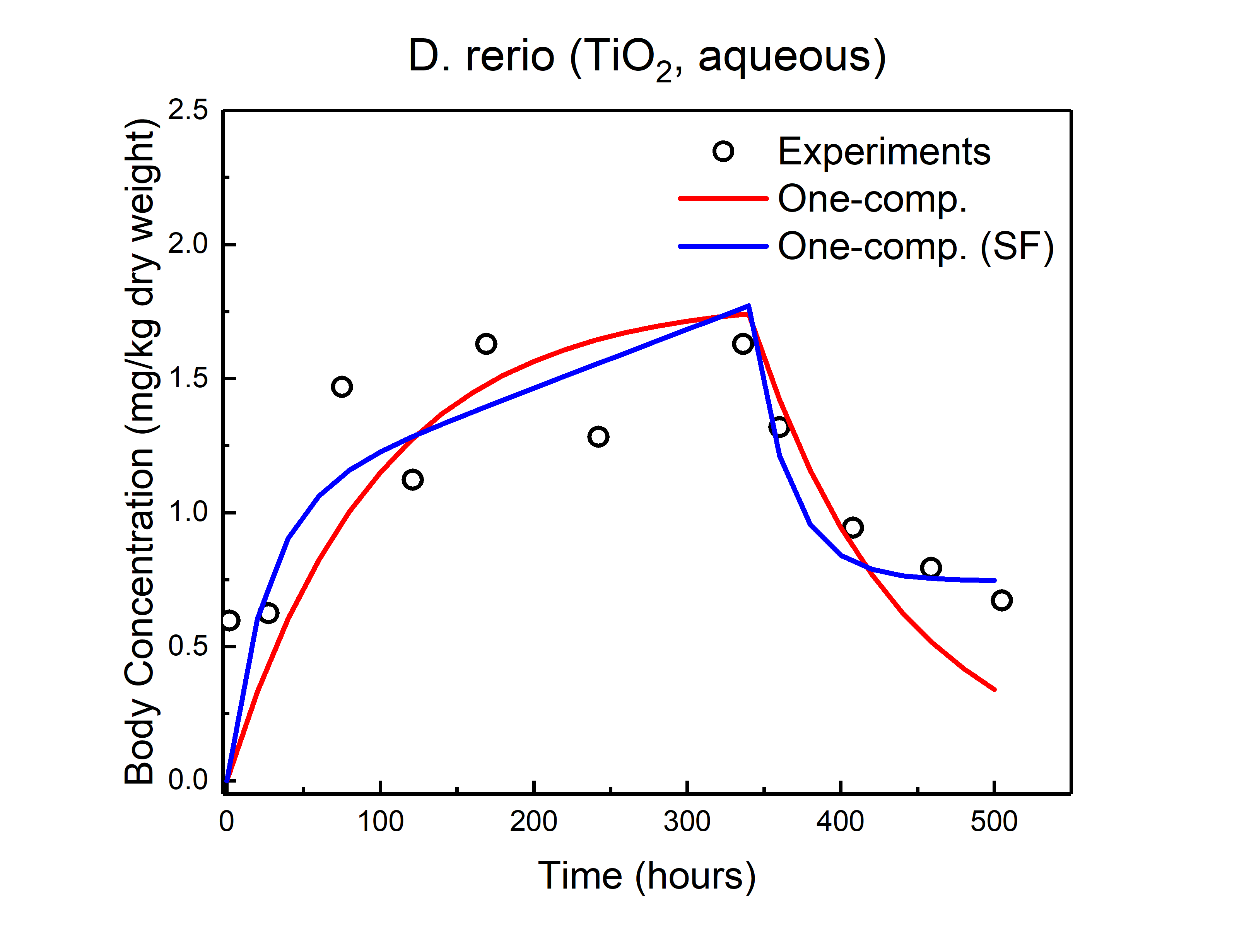


**Figure S8.** NanoBioAccumulate web application output with experimental data^6^ for the one compartment model and one compartment with a stored fraction (SF) model for modeling the kinetics of TiO_2_ in D. rerio exposed in aqueous environment.

# References

(1) Chen, Q.; Hu, X.; Yin, D.; Wang, R. Effect of subcellular distribution on nC60 uptake and transfer efficiency from Scenedesmus obliquus to Daphnia magna. *Ecotoxicology and Environmental Safety* **2016**, *128*, 213-221.

(2) Rivero Arze, A.; Manier, N.; Chatel, A.; Mouneyrac, C. Characterization of the nano–bio interaction between metallic oxide nanomaterials and freshwater microalgae using flow cytometry. *Nanotoxicology* **2020**, *14* (8), 1082-1095.

(3) Guo, X.; Dong, S.; Petersen, E. J.; Gao, S.; Huang, Q.; Mao, L. Biological uptake and depuration of radio-labeled graphene by Daphnia magna. *Environmental science & technology* **2013**, *47* (21), 12524-12531.

(4) Lv, X.; Yang, Y.; Tao, Y.; Jiang, Y.; Chen, B.; Zhu, X.; Cai, Z.; Li, B. A mechanism study on toxicity of graphene oxide to Daphnia magna: Direct link between bioaccumulation and oxidative stress. *Environmental pollution* **2018**, *234*, 953-959.

(5) Skjolding, L. M.; Kern, K.; Hjorth, R.; Hartmann, N.; Overgaard, S.; Ma, G.; Veinot, J.; Baun, A. Uptake and depuration of gold nanoparticles in Daphnia magna. *Ecotoxicology* **2014**, *23*, 1172-1183.

(6) Zhu, X.; Wang, J.; Zhang, X.; Chang, Y.; Chen, Y. Trophic transfer of TiO2 nanoparticles from daphnia to zebrafish in a simplified freshwater food chain. *Chemosphere* **2010**, *79* (9), 928-933.
